# Adenovirus vectors from various serotypes induce distinct cytokine profiles

**DOI:** 10.1186/1742-4690-9-S2-O15

**Published:** 2012-09-13

**Authors:** JE Teigler, M Iampietro, DH Barouch

**Affiliations:** 1 Division of Vaccine Research, Beth Israel Deaconess Medical Center, Boston, MA, USA

## Background

Adenovirus (Ad) vectors from various serotypes which differ markedly in their basic biology are being pursued as candidate HIV vaccines. However, the innate immune responses elicited by different Ad vectors remain poorly characterized. We therefore evaluated cytokine responses to Ad vector stimulation both in vitro in human PBMC and in vivo following vaccination of rhesus monkeys.

## Methods

Human PBMC were stimulated in vitro with 10^3^ vp/cell Ad5, Ad35, Ad26, Ad48, or Ad5/35 chimeric vectors. Rhesus monkeys were immunized with 3x10^10^ vp Ad5, Ad35, Ad26, Ad48, or Ad5HVR48. Cytokines in culture supernatant and serum from vaccinated monkeys were measured by luminex assays and ELISA.

## Results

Ad35 and Ad26 induced higher levels of antiviral and proinflammatory cytokines (e.g. IFNα2, IFNγ, IL-1β) compared to Ad5 in human PBMC (p<0.01, Kruskal-Wallis test; Dunn’s correction). Replacement of Ad5 fiber with that of Ad35 (Ad5f35) increased cytokine induction, while Ad35f5 displayed decreased stimulation, indicating the importance of fiber-receptor interactions for innate immune stimulation. Similarly, monkeys vaccinated with Ad35 or Ad26 also displayed markedly higher levels of antiviral and proinflammatory cytokines compared to Ad5 on day 1 post-vaccination (p<0.05, Mann-Whitney U test).

## Conclusion

These data demonstrate that CD46-uilizing Ad35 and Ad26 vectors induce profoundly different innate immune responses as compared to CAR-utilizing Ad5 vectors both in vitro and in vivo. These findings confirm that major biologic differences exist among Ad vectors and may help explain their different adaptive immune phenotypes.

